# A novel vascular health index: Using data analytics and population health to facilitate mechanistic modeling of microvascular status

**DOI:** 10.3389/fphys.2022.1071813

**Published:** 2022-12-06

**Authors:** Nithin J. Menon, Brayden D. Halvorson, Gabrielle H. Alimorad, Jefferson C. Frisbee, Daniel J. Lizotte, Aaron D. Ward, Daniel Goldman, Paul D. Chantler, Stephanie J. Frisbee

**Affiliations:** ^1^ Department of Medical Biophysics, Schulich School of Medicine & Dentistry, University of Western Ontario, London, ON, Canada; ^2^ Department of Epidemiology and Biostatistics, Schulich School of Medicine & Dentistry, University of Western Ontario, London, ON, Canada; ^3^ Department of Computer Science, Faculty of Science, University of Western Ontario, London, ON, Canada; ^4^ Lawson Health Research Institute, London, ON, Canada; ^5^ Department of Human Performance-Exercise Physiology, School of Medicine, West Virginia University, Morgantown, WV, United States; ^6^ Department of Pathology and Laboratory Medicine, Schulich School of Medicine & Dentistry, University of Western Ontario, London, ON, Canada

**Keywords:** microcirculation, vascular biology, metabolic disease, novel metrics, vascular health and disease

## Abstract

The study of vascular function across conditions has been an intensive area of investigation for many years. While these efforts have revealed many factors contributing to vascular health, challenges remain for integrating results across research groups, animal models, and experimental conditions to understand integrated vascular function. As such, the insights attained in clinical/population research from linking datasets, have not been fully realized in the basic sciences, thus frustrating advanced analytics and complex modeling. To achieve comparable advances, we must address the conceptual challenge of defining/measuring integrated vascular function and the technical challenge of combining data across conditions, models, and groups. Here, we describe an approach to establish and validate a composite metric of vascular function by comparing parameters of vascular function in metabolic disease (the obese Zucker rat) to the same parameters in age-matched, “healthy” conditions, resulting in a common outcome measure which we term the vascular health index (VHI). VHI allows for the integration of datasets, thus expanding sample size and permitting advanced modeling to gain insight into the development of peripheral and cerebral vascular dysfunction. Markers of vascular reactivity, vascular wall mechanics, and microvascular network density are integrated in the VHI. We provide a detailed presentation of the development of the VHI and provide multiple measures to assess face, content, criterion, and discriminant validity of the metric. Our results demonstrate how the VHI captures multiple indices of dysfunction in the skeletal muscle and cerebral vasculature with metabolic disease and provide context for an integrated understanding of vascular health under challenged conditions.

## Introduction

The study of vascular function under control or “healthy” conditions has been an intensive area of investigation across the translational research spectrum for many years. Operating in parallel have been investigations into how the defining parameters and integrated systems of vascular function are modified with the development of states of elevated disease risk or outright disease itself (Imig, 2020; Koenen et al., 2021), healthy aging (Zhou et al., 2021), or a variety of imposed experimental conditions (e.g., physical inactivity (Daniele et al., 2022), traumatic injury (Sandsmark et al., 2019), etc.). While these diverse efforts have provided us with a broad perspective for what constitutes vascular health and dysfunction, it has also created challenges for our ability to compare and integrate results across numerous research groups, animal models, and experimental conditions. Specifically, we continue to struggle with our ability to move from correlative measures and predictive biomarkers of vascular dysfunction to an understanding of integrated vascular function within the larger context of health and disease. Beyond gaining insight into markers of dysfunction and how they may frame our understanding of the impact of disease and elevated disease risk, it is increasingly important for us to be able to place the experimental results and datasets from different models of vasculopathy risk onto an appropriate spectrum of function. The general question to be considered is “how dysfunctional is the vasculature compared to ideal or to other compromised conditions?”.

When we consider the issues of developing an integrated perspective of vascular dysfunction, we encounter two categories of conceptual challenges: data-independent issues (i.e., comparability issues across models, research groups, and experimental approaches) and data-dependent issues (i.e., issues related to database content, statistical and analytical constraints, etc.). From the perspective of data-independency, we must consider conceptual challenges from a physiological perspective; specifically, how to define and quantify “vascular function” across a spectrum of conditions. This can include relative contributions of mediators of vascular function, the relative importance of these contributors in the spatial and temporal regulation of tissue perfusion, and even the targeting of interventions to achieve prodromic or postdromic prevention in specific conditions. Further, heterogeneity in models and approaches used must be considered such that differences between experimental designs undertaken by different research groups can be effectively compared.

From the perspective of data-dependency, there are several practical and technical challenges facing basic scientists from the perspectives of comparing datasets between conditions and research groups. These can include small sample sizes (as compared to population science or clinical cohort studies) and complex study designs that include highly specialized preparations, protocols or treatment regimes designed to address very specific research questions or hypotheses. For any given study, while a tremendous number of variables may be collected or generated, the traditional basic science paradigm typically limits the use of these data beyond their original intent, prohibiting the pooling or combining of data from different studies at different time points to address emerging questions. Consequently, the advances, insights, and knowledge realized in clinical and population research settings have not been realized by the basic sciences. This can include techniques and approaches from artificial intelligence/machine learning, “big data” techniques, and metanalyses, and linkage of datasets and sources across data types and time, from imaging data to electronic medical records to social services to birth registries and tissue banks. Additionally, basic sciences are further limited because there are no “secondary sources” of animal data that scientists can readily access—all data is, by necessity, primary data and each experiment can cost many thousands of dollars depending upon the animal model and specific performed procedures.

To realize the comparable benefits that data science has afforded the human subject and clinical research realms of the translational spectrum, basic scientists must develop approaches and tools that will allow them to consolidate data from multiple primary studies, collected at different levels of spatial and temporal resolution and across diverse experimental protocols. Further, they must also be able to consolidate many high-resolution data points into more encompassing constructs of “health,” “dysfunction,” or “disease” that allow for broader comparability. A goal is to transition from empirical evaluations of pathways and mechanisms toward a more complex analysis of integrated, holistic mechanistic models. In this manuscript, we describe an approach to establish and validate an integrated, composite metric that quantifies vascular dysfunction in elevated peripheral vascular disease (PVD) and cerebrovascular disease (CeVD) risk states as compared to vascular function in healthy, age-matched, control conditions. Once developed and validated, this metric can be used in mechanistic modeling to gain greater insight into the temporal and spatial development of vascular dysfunction within the context of physiological and pathophysiological systems. Finally, it is anticipated that the general approach to the development of such an index could be applied by other research groups to achieve similar goals in transitioning to more integrated, holistic mechanistic models. The primary objectives of this study are to develop an integrated vascular health index (VHI) for both the skeletal muscle and cerebral circulations and report on the presentation and validity of the metric.

## Materials and methods

Much of the data in the present manuscript have been published previously and these citations will be made at the appropriate points in the text. However, this manuscript represents the inclusion of both *de novo* experiments and analyses, previously unpublished results and analyses, and the pooling of data from previous studies for novel analyses. The present study serves as part of a larger goal of gaining additional insights from data collected from previous experiments and peer-reviewed, published studies by utilizing data science and more sophisticated analytic approaches, and mechanistic modeling. The protocols and specific methodology for the collection of the specific vascular phenotypes and the subsequent calculation of a Vascular Health Index (VHI) are summarized and fully referenced below.

### Animal model

All experiments and analyses described in this manuscript utilized male lean (LZR) and obese (OZR) Zucker rats. Animals were purchased from the supplier (Harlan/Envigo) at 6–7 weeks of age and were housed in an accredited animal care facility at the Medical College of Wisconsin, West Virginia University, or the University of Western Ontario, with *ad libitum* access to normal chow and water until the time of final usage unless otherwise noted. Following 1 week of acclimation, rats were placed into one of three groups until their final usage:

(1) Time control (LZR and OZR were housed without intervention and aged to a maximum of ∼20 weeks)
(2) Treadmill exercise (20 m/min, 5% incline, 60 min/day, 6 days/week; (Lemaster et al., 2018))
(3) Captopril (angiotensin converting enzyme inhibitor captopril (60 mg•kg^−1^•day^−1^); mixed with food (Frisbee et al., 2018))

At the time of final usage, each rat was deeply anesthetized with sodium pentobarbital (50 mg•kg^−1^ i.p.) and the trachea was intubated to maintain a patent airway. In all rats, a carotid artery and an external jugular vein were cannulated to measure arterial pressure and to infuse additional anesthetic, respectively, as necessary. At this time, an aliquot of blood was drawn from the jugular vein of each animal to be used for the subsequent determination of plasma metabolic/endocrine, oxidant stress, and inflammatory biomarker profiles using commercially available kits ((Frisbee et al., 2018)). All procedures followed approved IACUC protocols at each institution.

### Evaluation of vascular reactivity

The assessment of arteriolar reactivity from skeletal muscle was determined using the intramuscular continuation of the gracilis arteries, which were removed from each leg following the procedures in the neck (above). Subsequently, the rat was given a lethal overdose of pentobarbital anesthetic, followed by the removal of the head *via* decapitation. For the assessment of cerebrovascular reactivity, the middle cerebral arteries (MCA) were removed from their origin on the Circle of Willis following the removal of the brain from the skull. Both the gracilis muscle arterioles and the MCAs were doubly-cannulated and placed in a heated chamber (37°C) that allowed the vessel lumen and exterior to be perfused and superfused, respectively, with physiological salt solution (PSS; equilibrated with 21% O_2_, 5% CO_2_; 74% N_2_) from separate reservoirs (Fredricks et al., 1994a; Liu et al., 1997). Vessel diameter was measured using television microscopy and an on-screen video micrometer. Both vessels were extended to their *in situ* length and were equilibrated at 80% of the animal’s mean arterial pressure (Fredricks et al., 1994a; Liu et al., 1997).

In both gracilis muscle arterioles and MCAs, vascular reactivity was evaluated in response to application of acetylcholine (10^−9^ M–10^−6^ M) and sodium nitroprusside (10^−9^ M–10^−6^ M). In addition, the reactivity of isolated MCAs and gracilis muscle arterioles was assessed following hypoxic challenge with reduced PO_2_ (ΔPO_2_ from ∼135 mmHg [21% O_2_] – ∼45 mmHg [0% O_2_]) to assess endothelial function and dilator responses (Fredricks et al., 1994b).

The mechanical responses of isolated arterioles following pharmacological challenge with any of the agonists were fit with the following logistic equation:

$$y=\min+\left[\frac{\max-\min}{1+10^{\log EC_{50}-x}}\right]$$

where y represents the change in arteriolar diameter, “min” and “max” represent the lower and upper bounds, respectively, of the change in arteriolar diameter with increasing agonist concentration, x is the logarithm of the agonist concentration and $\log EC_{50}$ represents the logarithm of the agonist concentration ( x ) at which the response ( y ) is halfway between the lower and upper bounds.

### Evaluation of vascular wall mechanics

Following the experimental procedures for measuring *ex vivo* vascular reactivity for both MCA and gracilis arterioles, the perfusate and superfusate PSS were replaced with Ca^2+^-free PSS containing the metal ion chelators EDTA (0.03 mM) and EGTA (2.0 mM). Vessels were challenged with 10^−7^ M phenylephrine (gracilis arterioles) or serotonin (middle cerebral arteries) until all active tone was lost. Subsequently, intralumenal pressure within the isolated vessel was altered, in 20 mmHg increments, between 0 mmHg and 160 mmHg. To ensure that a negative intralumenal pressure was not exerted on the vessel, 5 mmHg was used as the “0 mmHg” intralumenal pressure point; all other values of intralumenal pressure were multiples of 20 mmHg up to 160 mmHg. After 5–7 min at each intralumenal pressure, the inner and outer diameter of the isolated vessel was determined.

All calculations of passive arteriolar wall mechanics (used as indicators of structural alterations to the individual microvessel) are based on those used previously (Baumbach and Hajdu, 1993; Brooks et al., 2018). Vessel wall thickness was calculated as:

$$WT=\frac{\left(OD-ID\right)}{2}$$

where WT represents wall thickness (μm) and OD and ID represent arteriolar outer and inner diameter, respectively (μm).

For the calculation of circumferential stress, intralumenal pressure was converted from mmHg to N/m^2^, where 1 mmHg = 1.334 × 10^2^ N/m^2^. Circumferential stress ( σ ) was then calculated as:

σ=PIL×ID2WT

where $P_{IL}$ represents the intralumenal pressure. Circumferential strain ( ε ) was calculated as:

ε=ID−ID5ID5

where ${ID}_{5}$ represents the internal arteriolar diameter at the lowest intralumenal pressure (i.e., 5 mmHg). The stress versus strain relationship from each vessel was fit (ordinary least squares analyses, r^2^ > 0.85) with the following exponential equation:

$$\sigma =\sigma_{5}e^{\beta \varepsilon }$$

where $\sigma_{5}$ represents circumferential stress at ${ID}_{5}$ and β is the slope coefficient describing arterial stiffness. Higher levels of β are indicative of increasing arterial stiffness (i.e., requiring a greater degree of distending pressure to achieve a given level of wall deformation; (Milnor, 1982; Baumbach and Hajdu, 1993)).

### Histological determination of skeletal muscle microvessel density

At the conclusion of the muscle contraction protocols, the gastrocnemius muscle was removed, rinsed in PSS and fixed in 0.25% formalin. Muscles were embedded in paraffin and cut into 5 μm cross sections. Sections were incubated with *Griffonia simplicifolia* I lectin (a general microvessel stain for all vessels <20 μm diameter), as described previously (Greene et al., 1990; Hansen-Smith et al., 1990). After exposure to lectin, sections were rinsed three times in PSS and were mounted on microscope slides with a water-soluble mounting medium (SP, ACCU-MOUNT 280, Baxter). Using epifluorescence microscopy, localization of labeled microvessels was performed with a Nikon E600 upright microscope with a ×20 objective lens (Plan Fluo phase NA 0.5). Excitation was provided by a 75 W Xenon Arc lamp through a Lambda 10–2 optical filter changer (Sutter Instrument Company, Novato, CA, United States) controlling a 595 nm excitation filter and a 615 nm emission filter. The microscope was coupled to cooled CCD camera (Micromax; Princeton Instruments Inc., Trenton, NJ, United States). From each gastrocnemius muscle, six individual cross sections were used for analysis, with six randomly selected regions within an individual cross section chosen for study and each region of study had an area of ∼1.47 × 10^5^ μm^2^ (Frisbee, 2003; Frisbee et al., 2014). All acquired images from individual sections were analyzed for number of microvessels and number of skeletal muscle fibers using MetaMorph Imaging software (Universal Imaging Co., Downingtown, PA, United States).

### Determination of cerebral cortex microvessel density

Following removal of the MCAs from the Circle of Willis on the base of the brain, the brain was placed within Tissue-Tek OCT compound and frozen. Brains were then sliced into 5 μm cross sections and where then stained using the established approach developed by Munzenmaier and Greene (Munzenmaier and Greene, 2006) using primary anti-CD-31 antibody. Under microscopy, localization of labeled microvessels was performed with a Nikon E600 upright microscope with a ×20 objective lens (Chantler et al., 2015). The microscope was coupled to cooled CCD camera (Micromax; Princeton Instruments Inc., Trenton, NJ, United States). Five nearby 1 mm^2^ images were taken from each of three sections in the frontal cortex of each brain, and the mean microvessel density within these 15 images was taken to represent cortical MVD in that animal. All acquired images from individual sections were analyzed for number of microvessels using MetaMorph Imaging software (Universal Imaging Co., Downingtown, PA, United States) (Chantler et al., 2015).

#### Development of the vascular health index

##### Determination of VHI characteristics:

In developing the VHI and ensuring it can effectively capture critical aspects of vascular structure and function, the following considerations were made:

(1) Given the many structural and functional differences between the cerebral and peripheral vasculature, a metric representing the health of their vasculature must be calibrated and calculated separately to yield a cerebral VHI and a peripheral VHI.
(2) The metric must be a composite measure that accounts for distinct aspects of vascular function and structure, such as vessel reactivity, vascular wall mechanics, and network characteristics.
(3) The metric will not be a predictive measure but instead will describe the relative state of the vasculature at a given time. Given this, there is no basis for establishing parameter weighting or coefficients for the different components within the composite metric. That is, all components are weighted equally.
(4) The use and calculation of such a metric needs to be practical and feasible insofar that the components of the metric need to be relatively easily collected in sufficient frequency to facilitate the actual quantitative determination of the metric in significant amounts, not just for our research group but other interested research teams as well (Baumbach and Hajdu, 1993).
(5) Given that the validity of a metric is the degree to which values from that metric represent the variable they intend to, we will need several forms of evidence to establish VHI as a valid estimator of vascular health. The specific forms of evidence/aspects of validity of concern are:
  (a) Face validity (Price et al., 2015)**:** the extent to which a metric appears to measure the construct of interest; are the parameters used in the development of the metric appropriate to the intention?
  (b) Content Validity (Price et al., 2015)**:** determines whether the index is appropriately representative of the aspects of the system being modeled. Does the content of the metric encompass the relevant aspects it is intended to estimate?
  (c) Criterion Validity (Price et al., 2015)**:** the extent to which the index responds in a manner that is consistent with general understanding and developed hypotheses and represents how well the value of the metric is indicative of the underlying theory of the system. Specifically, it is the extent to which values of a metric are correlated with other criterion variables that one would expect the measure to be correlated with.
  (d) Discriminant Validity (Price et al., 2015)**:** this determines the ability of the metric to distinguish between populations with differing levels of the underlying construct (vascular health). For example, our metric must be able to distinguish a population of obese Zucker rats from their lean counterparts as well as any effective treatment group that works to mitigate or minimize vascular dysfunction (like a population of obese Zucker rats under an exercise or drug regimen).

In evaluating the above criteria, we have developed a 3-component and 5-component version of both a cerebral VHI and peripheral VHI in Zucker rats. The rationale is that, for many of the data pooling opportunities across time and research groups, the use of a 3-component metric (composed of commonly collected measures) would allow for much larger sample sizes while still capturing the essential aspects of vascular health.

##### VHI parameter selection

Three fundamental aspects of healthy vasculature are the ability of resistance vessels to respond appropriately to vasoactive stimuli, the mechanics (i.e., the distensibility or stiffness) of the arteriolar wall, and the structure of the microvascular network from the perspective of a microvessel/capillary density within perfused tissue. Thus, to ensure face validity as well as content validity, the components of the VHI were selected to represent these differing major descriptors of vascular health. For the 3-component VHI, vascular responses to acetylcholine (reactivity), the slope ( β ) coefficient from the vascular wall stress versus strain relation (wall mechanics) and tissue microvessel density (network structure) were selected. For the 5-component VHI, these same parameters were used with the additional inclusion of two markers of vascular reactivity: the responses to sodium nitroprusside and to hypoxia.

#### Assessing the four aspects of validity

##### Face validity

**FIGURE 1:**
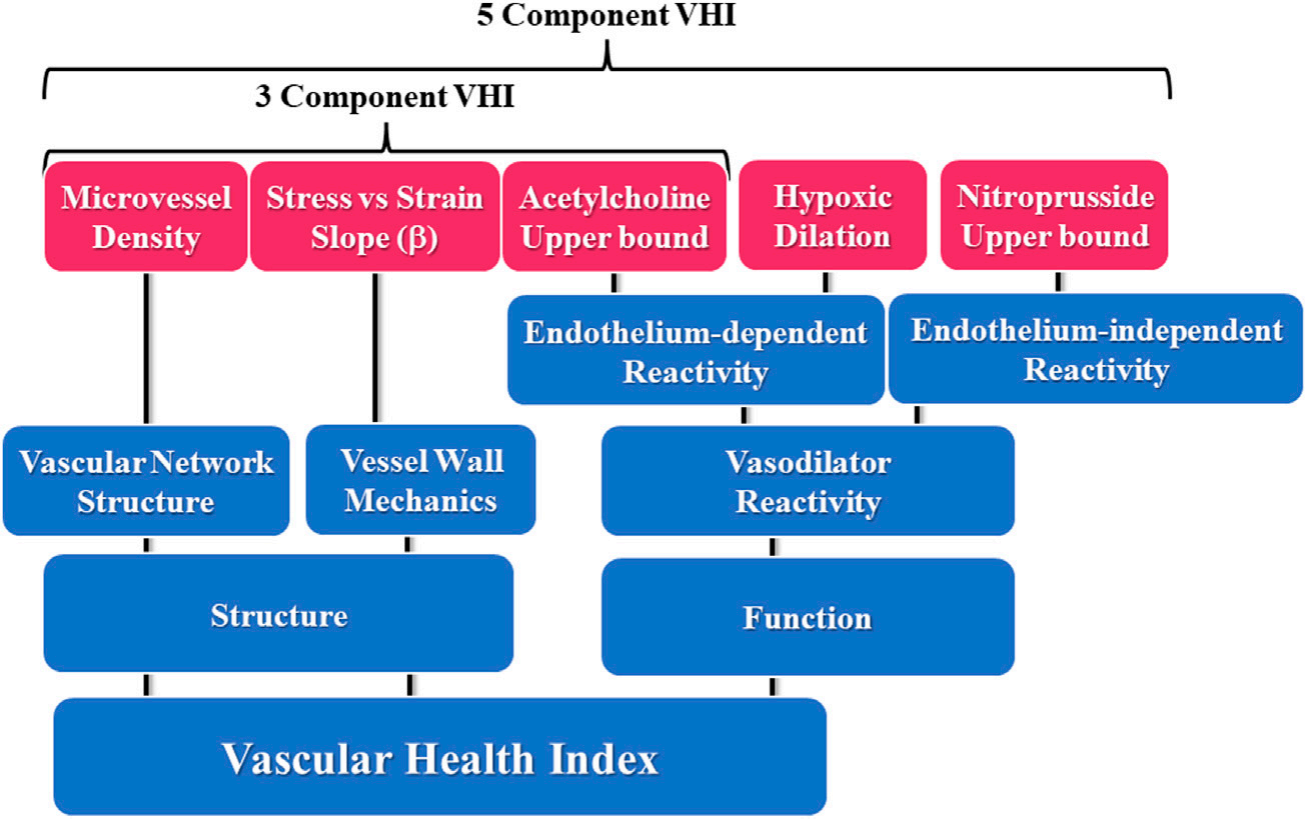
Schematic representation of the components contributing to the 5- and 3-parameter calculations for the Vascular Health Index (VHI), and their context within the scope of vascular structure and function. Please see text for details.

Being the weakest and least rigorous form of validity, face validity is often assessed informally. For our purposes with VHI, the assessment was made in the process of parameter selection for the components of the measure. This is represented in Figure 1, where the conceptual design summarizes the major aspects of vascular function used in the present study, where each of the domains are represented in the calculations of VHI.

##### Content validity

The content validity of the measure was ensured by clearly defining and restricting the components of “vascular health” to three major aspects and appropriately representing those aspects in the VHI. The three main aspects of vascular health are the reactivity of resistance vessels, arteriolar wall mechanics, and microvessel density within the tissue.

##### Criterion validity

In assessing criterion validity we need to identify a variable with which we would expect individual values of VHI in a given population to be correlated. For the present study, both plasma insulin and TNF-α concentrations were selected as the criterion with an expected correlation to VHI in a population of obese Zucker rats. To demonstrate criterion validity there needs to be a significant correlation between the insulin and TNF-α concentrations and the VHI of obese Zucker rats aged 7 weeks–20 weeks.

##### Discriminant validity

We will demonstrate the metric’s discriminant validity by distinguishing specific populations of Zucker rats using only their VHI values; specifically, populations of obese Zucker rats, obese Zucker rats that underwent a regular exercise regimen, obese Zucker rats that have been treated with an anti-hypertensive ACE inhibitor (captopril), and lean Zucker rats.

##### Construction of the measure

In constructing VHI and its calculation, the following general principles were used:

(1) The metric will be calculated at different age points (7, 10, 13, 15, 17, and 20 weeks old); meaning comparisons are done between lean and obese Zucker rats across ages of the animal and the resultant composite score can be considered independent of the specific age of the animals.
(2) With the “ideal” vascular health quantified in control rats, an animal experiencing an altered condition from this (e.g., elevated disease risk, interventional treatments, etc.) can then be quantitatively compared to this ideal standard of health. The simplest way to do this is to calculate a percentage-based score, where the measurement for the sick animal is expressed as a percentage (%) of the value for the age-matched healthy standard. Thus, the metric is interpreted as % of ideal vascular health.
(3) A single parameter, the VHI, is then calculated by averaging the component score (a percentage of ideal) across all 3 or 5 components in the index. This is repeated for both the cerebral and peripheral index. To calculate the index in control (i.e., healthy, untreated) animals the calculations were performed as outlined in Table 1. It should be noted that, with both age and disease risk, some variables are expected to increase as animals (e.g., vascular wall stiffness) become unhealthy whereas others decrease (e.g., endothelial function). This has been addressed in the calculations for the vascular wall stiffness component score by treating the relative increase above the associated standard of health value as a corresponding deficit to the component score (%).
(4) The metric will be a measure of “health,” where healthy animals (male, lean, untreated Zucker rats) that served as control animals in previous studies were used to define ideal vascular function (ideal standards for each component of VHI) at different age points. Therefore, the VHI of a given rat will represent any unhealthy deviation in the health of its vasculature. Given the use of data across multiple studies, the VHI of any age matched rat under an experimental condition will be calculated relative to the specific control (male, untreated lean Zucker rats) values of the original experiment.

**TABLE 1:** Calculations used for the determination of individual VHI Components in the present study.

Component	Expected deviation from LZR with disease risk	Formula used to Calculate VHI Component
Acetylcholine-induced Dilation (upper bound; μm)	Reduced (↓)	$\frac{Measurement\;}{Standard\;of\;health\;}\times 100$
Microvessel Density (#/mm^2^)	Reduced (↓)	$\frac{Measurement\;}{Standard\;of\;health\;}\times 100$
Circumferential Stress vs. Strain Slope Coefficient (β)	Increased (↑)	$100+\left(100-\left[\frac{Measurement\;}{Standard\;of\;health\;}\times 100\right]\right)$
Sodium Nitroprusside-induced Dilation (upper bound; μm)	Reduced (↓)	$\frac{Measurement\;}{Standard\;of\;health\;}\times 100$
Hypoxic Dilation (μm)	Reduced (↓)	$\frac{Measurement\;}{Standard\;of\;health\;}\times 100$

## Results

Table 2 summarizes the samples sizes of all animal groups, at all ages, used in the present study. The baseline characteristics of the animals used in the present study, at each age, are summarized in Table 3. Tables 4, 5 present the raw data describing the different VHI components used in the present study for the peripheral (skeletal muscle) and cerebral vasculature, respectively. The aggregate values of skeletal muscle and cerebral VHI are summarized in Table 6.

**TABLE 2:** Animal numbers used in the present study. Data are presented for each group of animals and at each age for both the peripheral and cerebral vascular health index (VHI) calculations.

Animal group	Age (Weeks)	Peripheral VHI “n”		Cerebral VHI “n”	
		5 Component	3 Component	5 Component	3 Component
OZR	7	16	36	8	20
	10	16	30	8	8
	13	16	36	8	14
	17	28	28	8	12
	20	0	6	8	8
	Total	76	136	40	62
OZR + Exercise	7	7	7	—	—
	10	7	7	—	—
	13	7	7	—	—
	17	7	23	—	—
	20	—	—	—	—
	Total	28	44	—	—
OZR + Captopril	7	4	4	—	6
	10	4	4	—	—
	13	4	4	—	6
	17	4	4	—	6
	20	—	—	—	—
	Total	16	16	—	18
LZR	7	25	37	10	22
	10	23	29	10	10
	13	24	36	10	16
	17	22	42	10	12
	20	—	6	11	11
	Total	94	150	51	71

**TABLE 3:** Baseline characteristics of animals used in the present study.

Variable	Group	7 weeks	10 weeks	13 weeks	17 weeks	20 weeks
Mass (g)	LZR	149.7 ± 1.5^†^	243.0 ± 2.1^†^	307.2 ± 2.0^†^	357.5 ± 1.5^†^	374.3 ± 2.6^†^
	OZR	233.9 ± 1.8*	409.4 ± 2.6*	512.3 ± 3.2*	682.3 ± 2.5*	741.8 ± 11.0*
	OZR-Exer	187.3 ± 27.2*^†^	291.3 ± 29.7*^†^	359.3 ± 41.1*^†^	441.0 ± 53.3*^†^	—
	OZR-Cap	239.0 ± 2.7*	409.0 ± 4.5*	515.3 ± 4.6*	624.3 ± 8.6*	—
Insulin (ng/ml)	LZR	1.0 ± 0.1^†^	1.2 ± 0.1^†^	1.3 ± 0.1^†^	1.1 ± 0.1^†^	1.5 ± 0.1^†^
	OZR	3.5 ± 0.1*	5.0 ± 0.1*	7.6 ± 0.2*	7.8 ± 0.1*	10.8 ± 0.6*
	OZR-Exer	1.7 ± 0.4*^†^	2.4 ± 0.8*^†^	3.6 ± 1.5*^†^	4.3 ± 1.9*^†^	—
	OZR-Cap	3.5 ± 0.2*	3.7 ± 0.3*^†^	5.4 ± 0.3*^†^	6.7 ± 0.2*^†^	—
Glucose (mg/dl)	LZR	93.7 ± 1.1†	98.4 ± 1.1^†^	100.9 ± 1.1^†^	100.2 ± 1.4^†^	104.7 ± 2.0^†^
	OZR	99.7 ± 1.4*	118.6 ± 3.4*	138.6 ± 2.7*	179.1 ± 1.2*	182.6 ± 2.7*
	OZR-Exer	93.5 ± 5.4	94.0 ± 5.0^†^	105.0 ± 6.6^†^	119.0 ± 12.1*^†^	—
	OZR-Cap	99.0 ± 3.5*	100.0 ± 2.5^†^	123.5 ± 2.5*^†^	137.5 ± 5.3*^†^	—
N-tyr (ng/dl)	LZR	9.2 ± 0.3^†^	10.4 ± 0.3^†^	12.8 ± 0.3^†^	16.5 ± 0.6^†^	18.2 ± 1.0†
	OZR	15.0 ± 0.4*	24.8 ± 0.4*	45.4 ± 1.1*	51.7 ± 0.9*	59.6 ± 1.0*
	OZR-Exer	10.3 ± 0.7^†^	11.8 ± 1.3^†^	19.0 ± 4.7*^†^	20.5 ± 6.3*^†^	—
	OZR-Cap	13.0 ± 0.6*^†^	16.0 ± 0.7*^†^	24.5 ± 0.3*^†^	34.0 ± 0.9*^†^	—
TNF-α (pg/ml)	LZR	1.7 ± 0.1^†^	2.0 ± 0.1^†^	2.4 ± 0.2^†^	2.2 ± 0.1^†^	2.6 ± 0.2^†^
	OZR	4.5 ± 0.2*	8.6 ± 0.3*	10.9 ± 0.2*	8.1 ± 0.3*	13.3 ± 0.4*
	OZR-Exer	1.8 ± 0.3^†^	2.8 ± 0.8^†^	3.3 ± 1.0*†	3.5 ± 1.2*^†^	—
	OZR-Cap	3.5 ± 0.3*^†^	6.5 ± 0.7*^†^	7.0 ± 0.9*†	9.5 ± 0.7*^†^	—

*p < 0.05 vs. LZR at that age; ^†^ *p* < 0.05 vs. OZR at that age.

**TABLE 4:** Peripheral (skeletal muscle) vascular component data, presented as mean ± SE, for the animal groups of the present study across all age.

Component	Group	7 weeks	10 weeks	13 weeks	17 weeks	20 weeks
Acetylcholine Dilation (μm)	LZR	119.2 ± 1.1	125.0 ± 1.1^†^	129.3 ± 1.2^†^	136.5 ± 2.0^†^	139.9 ± 1.6^†^
	OZR	115.3 ± 1.9	118.4 ± 3.2*	120.5 ± 2.8*	122.6 ± 2.9*	119.3 ± 3.3*
	OZR-Exer	117.6 ± 2.6	127.3 ± 2.2^†^	131.7 ± 2.3^†^	129.6 ± 3.4*	—
	OZR-Cap	118.3 ± 2.7	117.0 ± 2.1*	124.8 ± 3.9	124.3 ± 1.9*	—
Nitroprusside Dilation (μm)	LZR	128.8 ± 0.9	130.6 ± 1.0	132.9 ± 1.2	140.3 ± 2.2	140.7 ± 1.4
	OZR	127.7 ± 2.2	131.5 ± 3.3	134.1 ± 2.6	139.8 ± 3.7	135.6 ± 4.8
	OZR-Exer	127.0 ± 2.7	132.0 ± 1.8	135.7 ± 2.3	134.4 ± 3.8	—
	OZR-Cap	130.8 ± 3.2	134.3 ± 1.5*	138.5 ± 3.4*	134.0 ± 2.6*	—
Microvessel Density (#/mm^2^)	LZR	809.9 ± 13.9	810.4 ± 13.1	811.8 ± 11.9^†^	863.1 ± 4.6^†^	823.0 ± 9.1^†^
	OZR	805.4 ± 25.2	782.4 ± 26.1	706.9 ± 16.9*	656.3 ± 20.5*	635.9 ± 11.6*
	OZR-Exer	881.4 ± 10.8*^†^	874.6 ± 14.6*^†^	832.3 ± 21.5^†^	869.4 ± 17.3^†^	—
	OZR-Cap	833.3 ± 12.3	813.3 ± 5.5	764.0 ± 8.0*^†^	730.5 ± 16.6*^†^	—
Hypoxic Dilation (μm)	LZR	120.8 ± 1.1	125.0 ± 1.0^†^	128.9 ± 1.2^†^	135.3 ± 1.8^†^	135.8 ± 2.0^†^
	OZR	117.0 ± 2.2	118.1 ± 3.1*	119.6 ± 3.1*	121.6 ± 4.1*	118.4 ± 2.1*
	OZR-Exer	121.1 ± 2.3	126.6 ± 2.3^†^	129.6 ± 2.5^†^	125.6 ± 4.8*	—
	OZR-Cap	119.5 ± 3.0	119.5 ± 2.3*	126.8 ± 3.2^†^	123.0 ± 4.0*	—
Stress vs. Strain β	LZR	2.6 ± 0.1	2.6 ± 0.1^†^	2.8 ± 0.1^†^	3.1 ± 0.1^†^	3.2 ± 0.1†
	OZR	2.4 ± 0.3	3.4 ± 0.5*	4.1 ± 0.6*	6.2 ± 0.4*	5.7 ± 0.7*
	OZR-Exer	3.7 ± 0.3*	3.5 ± 0.3*	3.8 ± 0.4*	4.0 ± 0.3*^†^	—
	OZR-Cap	3.2 ± 0.2*	3.5 ± 0.3*	4.2 ± 0.2*	4.8 ± 0.3*^†^	—

*p < 0.05 vs. LZR at that age; ^†^ *p* < 0.05 vs. OZR at that age.

**TABLE 5:** Cerebral vascular component data, presented as mean ± SE, for the animal groups of the present study across all age.

Component	Group	7 weeks	10 weeks	13 weeks	17 weeks	20 weeks
Acetylcholine Dilation	LZR	135.2 ± 1.3^†^	144.1 ± 0.9^†^	151.8 ± 0.8^†^	155.0 ± 1.4^†^	163.0 ± 1.1^†^
	OZR	122.4 ± 1.3*	128.4 ± 1.0*	125.1 ± 0.8*	122.9 ± 1.7*	119.8 ± 2.5*
	OZR-Exer	—	—	—	—	—
	OZR-Cap	137.5 ± 0.7	—	139.0 ± 0.7*	132.5 ± 2.4*	—
Nitroprusside Dilation	LZR	142.7 ± 1.2^†^	151.4 ± 0.6^†^	159.1 ± 1.2^†^	158.6 ± 1.1^†^	163.4 ± 1.1^†^
	OZR	131.1 ± 1.5*	135.3 ± 1.5*	142.1 ± 1.3*	139.8 ± 1.5*	138.5 ± 2.1*
	OZR-Exer	—	—	—	—	—
	OZR-Cap	—	—	—	—	—
Microvessel Density	LZR	290.0 ± 1.9^†^	293.0 ± 2.1^†^	303.8 ± 1.2^†^	313.6 ± 1.4^†^	319.6 ± 1.0^†^
	OZR	274.0 ± 3.2*	270.1 ± 2.6*	255.8 ± 2.6*	249.1 ± 2.1*	242.0 ± 1.9*
	OZR-Exer	—	—	—	—	—
	OZR-Cap	337.3 ± 2.7*^†^	-	332.0 ± 1.7*^†^	310.3 ± 2.2^†^	—
Hypoxic Dilation	LZR	132.1 ± 1.2^†^	140.2 ± 0.7^†^	149.5 ± 0.9^†^	152.6 ± 1.5^†^	159.8 ± 0.9^†^
	OZR	122.6 ± 1.6*	129.1 ± 1.0*	127.1 ± 0.8*	123.5 ± 1.1*	120.6 ± 2.4*
	OZR-Exer	—	—	—	—	—
	OZR-Cap	—	—	—	—	—
Stress vs. Strain β	LZR	1.6 ± 0.1	1.7 ± 0.1^†^	1.8 ± 0.1^†^	2.0 ± 0.1^†^	2.2 ± 0.1^†^
	OZR	1.8 ± 0.1	2.1 ± 0.1*	2.8 ± 0.2*	4.0 ± 0.2*	5.4 ± 0.1*
	OZR-Exer	—	—	—	—	—
	OZR-Cap	2.5 ± 0.1*†	—	3.3 ± 0.1*^†^	4.7 ± 0.1*^†^	—

*p < 0.05 vs. LZR at that age; ^†^ *p* < 0.05 vs. OZR at that age.

**TABLE 6:** Calculated Vascular Health Index (VHI) for each group of animals at each age within the present study.

Animal group	Age (Weeks)	Peripheral VHI “n”		Cerebral VHI “n”	
		5 Component	3 Component	5 Component	3 Component
OZR	7	101.5 ± 1.0	100.8 ± 1.2	91.3 ± 1.1*	92.8 ± 0.8*
	10	92.7 ± 1.7*	88.3 ± 1.9*	87.8 ± 0.8*	85.9 ± 1.2*
	13	86.6 ± 2.1*	79.6 ± 2.2*	76.6 ± 1.6*	75.4 ± 2.4*
	17	70.2 ± 0.9*	63.4 ± 1.3*	64.7 ± 1.8*	60.1 ± 3.1*
	20	—	42.2 ± 1.6*	52.0 ± 1.3*	33.2 ± 2.4*
	Mean	84.2 ± 1.3*	82.2 ± 1.6*	74.5 ± 2.4*	71.4 ± 2.7*
OZR + Exercise	7	97.2 ± 1.9	93.5 ± 3.1^†^	—	—
	10	97.1 ± 2.9	94.3 ± 4.6	—	—
	13	94.8 ± 4.1^†^	90.9 ± 5.7*^†^	—	—
	17	94.8 ± 3.7*^†^	86.3 ± 3.4*^†^	—	—
	20	—	—	—	—
	Mean	95.9 ± 1.6*^†^	89.7 ± 2.9*^†^	—	—
OZR + Captopril	7	99.9 ± 2.1	97.3 ± 2.1	—	95.6 ± 0.6*^†^
	10	92.9 ± 1.7*	88.7 ± 2.6*	—	—
	13	89.3 ± 0.9*	81.9 ± 1.1*	—	89.6 ± 1.4*^†^
	17	84.9 ± 1.4*^†^	76.8 ± 2.3*^†^	—	68.9 ± 0.6*^†^
	20	—	—	—	—
	Mean	88.7 ± 1.6*	86.2 ± 2.2*	—	84.7 ± 2.8*^†^

*p < 0.05 vs. LZR at that age; ^†^ *p* < 0.05 vs. OZR at that age.

### Peripheral vascular health index

**FIGURE 2:**
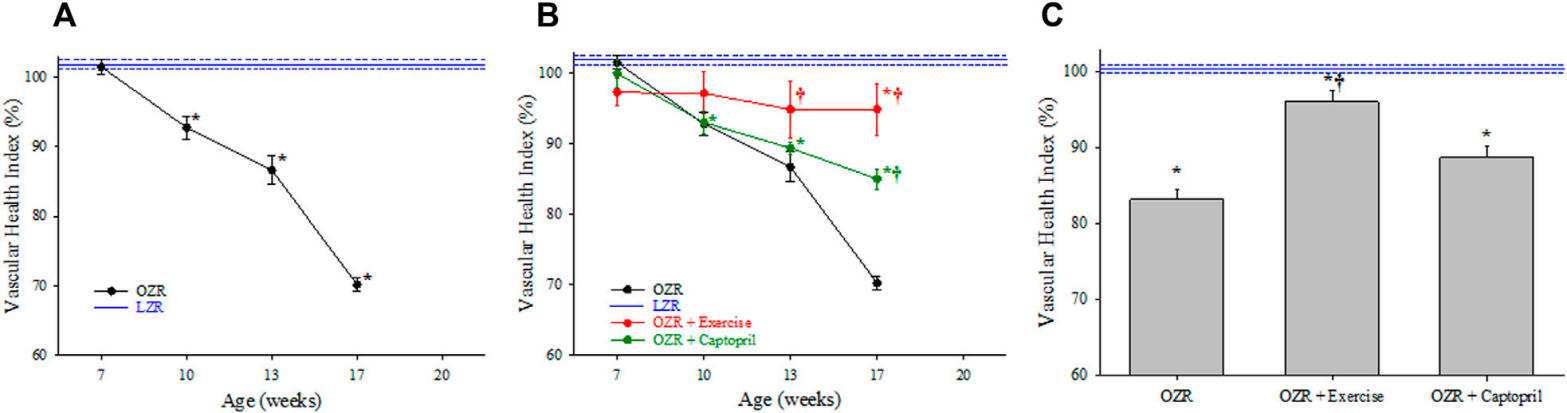
Data describing the 5-parameter determination of Vascular Health Index (VHI) within the peripheral/skeletal muscle microcirculation. Data (mean ± SE) are presented for OZR over the age ranges of the present study (Panel **(A)**) or the impact of chronic interventions with either exercise or captopril administration (Panel **(B)**). Panel **(C)** presents the aggregate VHI from the different animal groups where all ages have been compiled into one data point. VHI from LZR is set to 100%, by definition. *p < 0.05 vs. LZR at that age; ^†^ *p* < 0.05 vs. untreated OZR at that age. Please see text for details.

Using the 5 component analyses described above, Figure 2A summarizes the changes to the VHI in the peripheral microcirculation of OZR as compared to the control level of function in LZR (defined as 100%). With the progressive development of metabolic disease in OZR, there was a steady and significant decline in the magnitude of VHI throughout the age ranges used in the present study, culminating at 17 weeks of age. Figure 2B presents the impact of two chronic interventions, treatment with the anti-hypertensive drug captopril and daily treadmill exercise of moderate intensity on VHI in OZR. Both interventions were effective at significantly improving skeletal muscle VHI as compared to levels determined in untreated OZR, with chronic exercise resulting in a larger improvement to VHI as compared to that with anti-hypertensive therapy. Panel C presents the aggregate VHI score (i.e., all ages collapsed into one measurement of VHI) for OZR under control conditions and with chronic exercise and captopril interventions. While the aggregate VHI in OZR was significantly reduced as compared to LZR, the interventions with chronic exercise or captopril treatment resulted in significant improvements to aggregate VHI.

**FIGURE 3:**
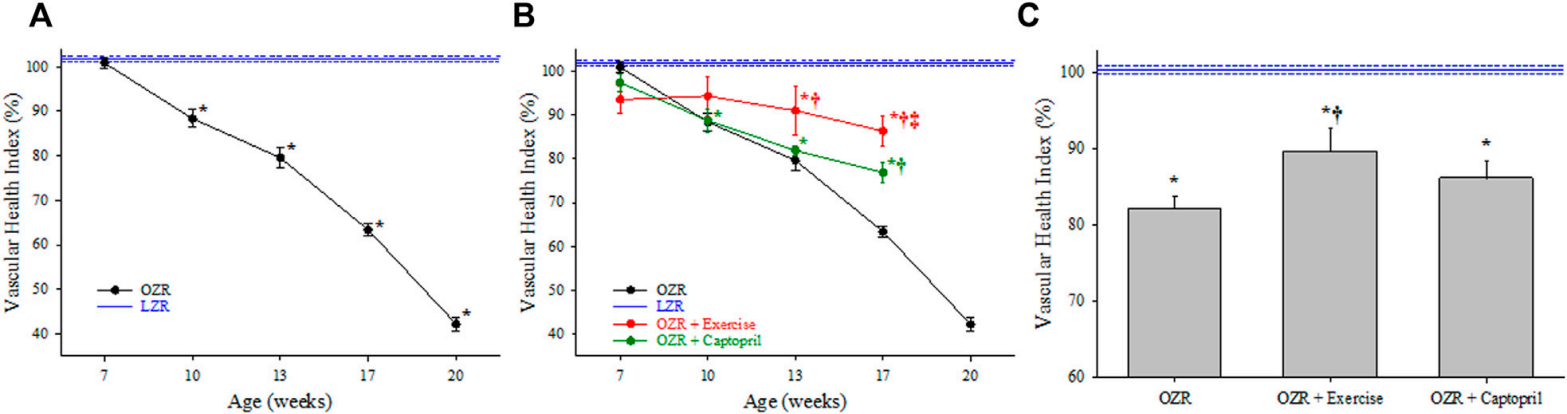
Data describing the 3-parameter determination of Vascular Health Index (VHI) within the peripheral/skeletal muscle microcirculation. Data (mean ± SE) are presented for OZR over the age ranges of the present study (Panel **(A)**) or the impact of chronic interventions with either exercise or captopril administration (Panel **(B)**). Panel **(C)** presents the aggregate VHI from the different animal groups where all ages have been compiled into one data point. VHI from LZR is set to 100%, by definition. *p < 0.05 vs. LZR at that age; ^†^ *p* < 0.05 vs. untreated OZR at that age. ^‡^ *p* < 0.05 vs. OZR + captopril at that age. Please see text for details.

Figure 3A, panel A presents the results of the 3-component analyses on skeletal muscle VHI in OZR with increasing age. While a similar trend was determined in terms of the decay in VHI in control OZR with the development of metabolic disease versus LZR as compared to that for the 5 component analyses, the calculated severity of the impairment was greater, reaching 40% of control LZR levels by 20 weeks of age. As presented in Panel B, chronic treatment with either captopril or treadmill exercise significantly improved VHI outcomes in OZR as compared to the untreated condition, with exercise being significantly more effective than captopril treatment. As presented in Panel C, chronic intervention with exercise improved VHI from levels determined in untreated OZR, although this effect was not significant with captopril treatment alone.

### Cerebral vascular health index

**FIGURE 4:**
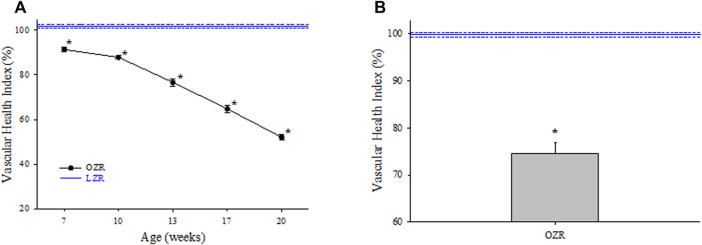
Data describing the 5-parameter determination of Vascular Health Index (VHI) within the cerebral microcirculation. Data (mean ± SE) are presented for OZR over the age ranges of the present study (Panel **(A)**). Panel **(B)** presents the aggregate VHI from OZR where all ages have been compiled into one data point. VHI from LZR is set to 100%, by definition. *p < 0.05 vs. LZR at that age. Please see text for details.

Using the 5-component calculation for VHI, the changes in cerebral microvascular health in OZR are presented in Figure 4. As compared to responses in control LZR, there was a progressive and significant reduction in cerebral VHI in OZR throughout the age range of the present study, reaching a maximum impairment of ∼50% from that determined in LZR. It is noted that a significant impairment to cerebral VHI in OZR was already present at the earliest age range (7 weeks), where VHI was already reduced by ∼10%, despite the severity of the evolving metabolic disease still being relatively mild (Table 3). The aggregate VHI for OZR across all ages is presented in Panel B, where it was significantly reduced from that calculated in untreated LZR.

**FIGURE 5:**
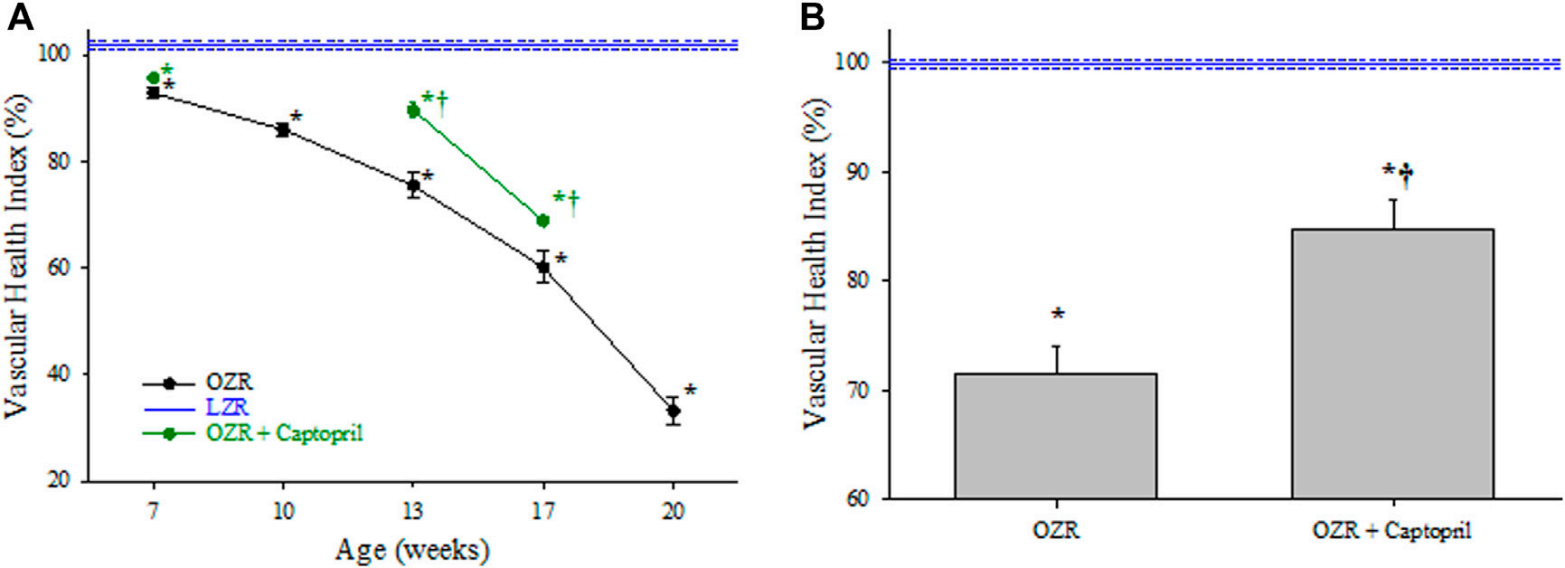
Data describing the 3-parameter determination of Vascular Health Index (VHI) within the cerebral microcirculation. Data (mean ± SE) are presented for OZR over the age ranges of the present study with and without chronic intervention with captopril administration (Panel **(A)**). Panel **(B)** presents the aggregate VHI from OZR, with and without captopril administration, where all ages have been compiled into one data point. VHI from LZR is set to 100%, by definition. *p < 0.05 vs. LZR at that age; ^†^ *p* < 0.05 vs. untreated OZR at that age. Please see text for details.

Figure 5 presents the changes to cerebral VHI using the 3-component analyses in OZR as compared to untreated LZR. The severity of the calculated impairment to cerebrovascular health was greater in OZR using the 3-parameter approach vs. the 5-component calculation, reaching only 30% of control VHI in LZR by 20 weeks of age. Also presented in Figure 5 are the results of chronic intervention with captopril on cerebral VHI, which resulted in a significant improvement to VHI as compared to that in untreated OZR at both 13 and 17 weeks of age. Panel B presents the data describing the aggregate cerebral VHI for OZR under control conditions and in response to chronic intervention with captopril. While VHI in untreated OZR was significantly reduced as compared to that in untreated LZR, chronic captopril treatment significantly improved VHI in OZR.

### Criterion validity

**FIGURE 6:**
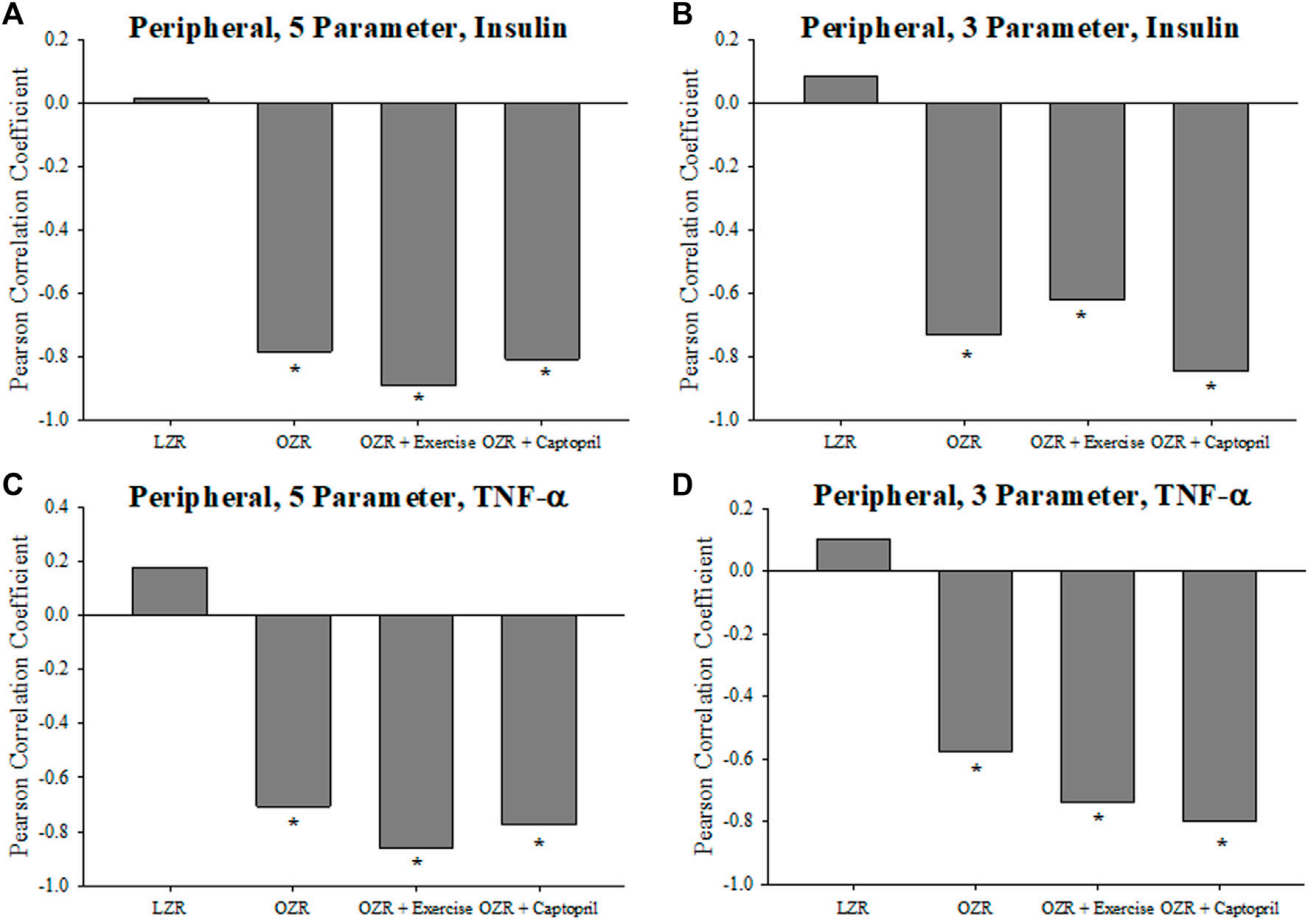
Data (mean ± SE) describing the criterion validity between plasma insulin or TNF-α and the skeletal muscle Vascular Health Index (VHI) in the present study. The Pearson Correlation Coefficient between insulin and VHI in LZR and OZR are summarized in Panels **(A)** (5-parameter VHI) and **(B)** (3-parameter VHI). Panels **(C)** (5-parameter VHI) and **(D)** (3-parameter VHI) present the Pearson Correlation Coefficient between plasma TNF-α and skeletal muscle VHI in LZR and OZR. Also presented are the correlations between plasma insulin or TNF-α and VHI in OZR following intervention with chronic exercise or captopril treatment. *p < 0.05 vs. LZR at that age; ^†^ *p* < 0.05 vs. OZR at that age. Please see text for details.

Figure 6 presents the criterion validity for the calculations of the peripheral VHI in the present study. In the case of the 5-component (Figure 6A) and the 3-component (Figure 6B) VHI, the plasma insulin concentration from the OZR groups were consistently and significantly negatively correlated with the vascular health index. This correlation reflects the strong tendency for OZR with a higher VHI to have the lowest insulin resistance. This similar pattern in also demonstrated for the 5-component (Figure 6C) and 3-component (Figure 6D) for the correlation between plasma TNF-α and VHI in OZR where higher levels of VHI were correlated with low levels of chronic inflammation in these animals.

**FIGURE 7:**
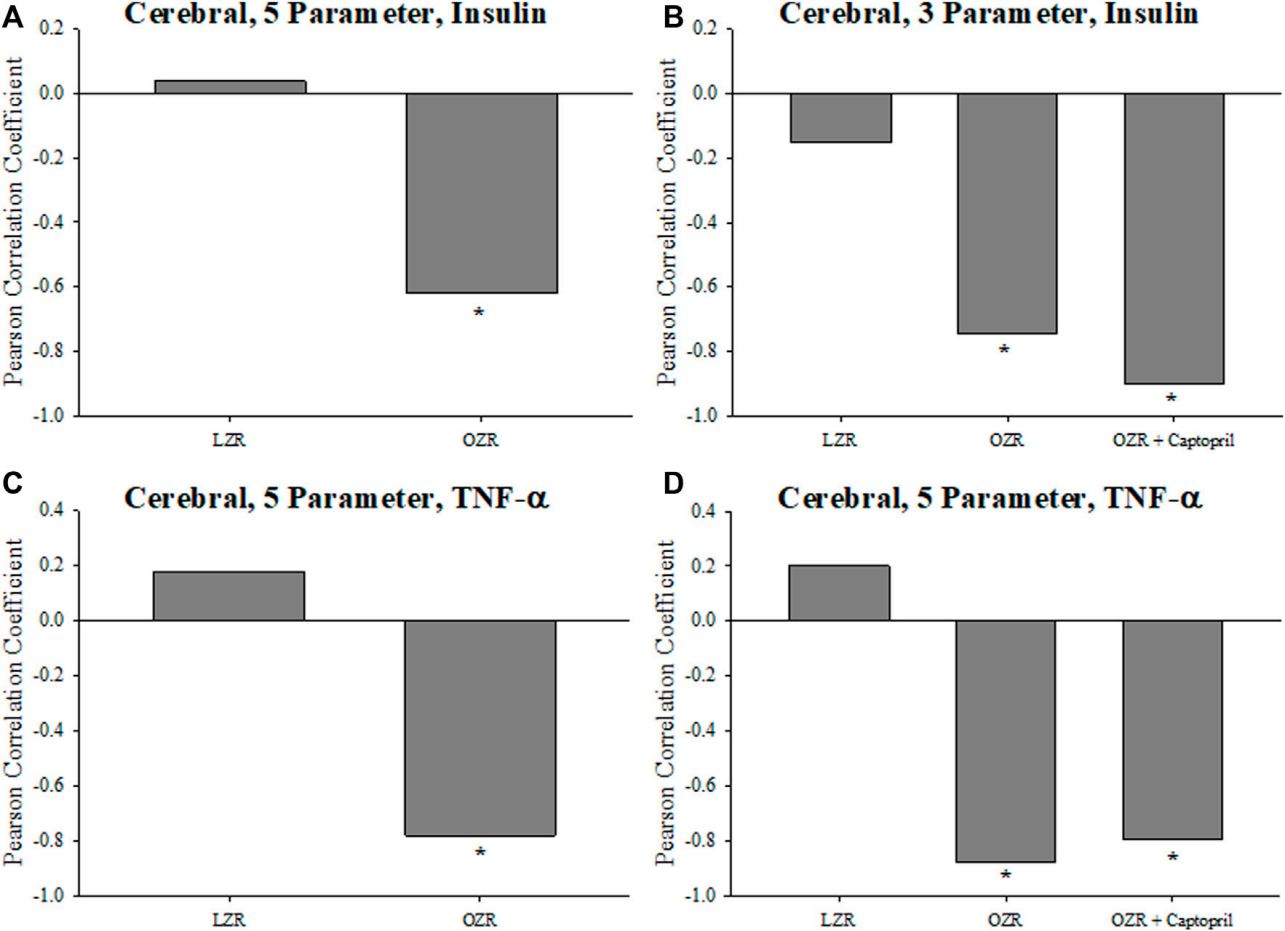
Data (mean ± SE) describing the criterion validity between plasma insulin or TNF-α and the cerebral Vascular Health Index (VHI) in the present study. The Pearson Correlation Coefficient between insulin and VHI in LZR and OZR are summarized in Panels **(A)** (5-parameter VHI) and **(B)** (3-parameter VHI). Panels **(C)** (5-parameter VHI) and **(D)** (3-parameter VHI) present the Pearson Correlation Coefficient between plasma TNF-α and cerebral VHI in LZR and OZR. Also presented are the correlations between plasma insulin or TNF-α and VHI in OZR following intervention with chronic captopril treatment. *p < 0.05 vs. LZR at that age; ^†^ *p* < 0.05 vs. OZR at that age. Please see text for details.

The criterion validity for the cerebral VHI calculations is summarized in Figure 7, where Figures 7A,B present the 5- and 3-component correlations with plasma insulin concentration, respectively. In both cases, the Pearson correlation coefficient was strongly negatively associated with VHI in OZR under untreated conditions and following chronic treatment with captopril. This same relationship was also evident for the relation between 5- (Figure 7C) and 3-parameter (Figure 7D) calculations of VHI and chronic inflammation, where the index was strongly and negatively correlated with TNF-α.

## Discussion

The overall purpose for the present study was to describe an approach for developing a comprehensive metric of integrated vascular/microvascular function that is not only a valid representation of vascular health, but that also allows for the pooling of data across multiple studies to gain greater inferential power. Subsequently, we calculated this metric using our original raw data in both the skeletal muscle (peripheral) and cerebral vasculature using a 3- and 5-component version of the VHI. Finally, we demonstrated the face, content, criterion and discriminant validity of the VHI. The primary result of this study is a validated score of integrated vascular function that can be used for more complex analytic and mechanistic modeling.

An initial question to be addressed is the utility of the VHI as it relates to an understanding of vasculopathy. When one considers a novel metric for assessing an integrated biological system, it is important to understand how the metric contributes beyond that of the individual parameters contributing to its calculation. Within the context of the present study, this is reflected as determining the superiority of VHI over individual analysis and interpretation of vascular reactivity, vascular wall mechanics and microvessel density on an individual basis. While it is certainly clear that individual metrics can provide a high-resolution inference into specific questions or hypotheses, they can be more limited in terms of understanding how complex, integrated outcomes are manifest (Lemaster et al., 2017a), thus requiring the development of a more holistic measurement. Further, the question of “is one approach better than the other?”, may not be appropriate. In reality, multiple measures should be acting as complementary approaches where the whole may be greater than the sum of the parts. As a clinical example, while individual measurements such as obesity, smoking and blood pressure can be used to better understand a person’s cardiovascular disease risk status, the Framingham Risk Score provides a context that cannot be provided by the individual parameters it is comprised of in isolation. A comparable situation exists in the current study with the individual markers of vasculopathy and the VHI, merely for basic science research.

It is also important to note that for the development of VHI, we have included no parameter weighting, which is frequently used with other integrated metrics (such as Framingham). This lack of parameter weighting reflects two issues. First, there is no clear *a priori* rationale for introducing parameter weighting at this time. Specifically, there is no clear evidence of the relative importance of vascular reactivity, wall mechanics or microvessel density in terms of contributions to health outcomes. While there is broad agreement that all are important and contribute, there is no consensus as to their rank ordering of importance. Second, to introduce parameter weighting at this time would reflect the use of a regression-based approach that was designed to predict an outcome. That is not the purpose of the VHI in this context (it makes no outcome predictions) and, as such, a regression-based approach was rejected in favor of the analytical approach described above.

An encompassing issue that should be considered is the selection of the different parameters that contribute to the VHI (Effros et al., 1981). In the present study, and as presented in Figure 1, we elected to split our analyses for either skeletal muscle or cerebral VHI into both 5- and 3-parameter calculations. At the initial assessment, it was decided that indicators of endothelium-dependent and independent dilation will be important as markers of vascular reactivity. These were encompassed through acetylcholine- and hypoxia-induced dilation for endothelium-dependency in both tissues and sodium nitroprusside-induced dilation for endothelium-independency. The use of acetylcholine and/or hypoxia as a dilator stimulus also allows for a discrimination between dilator responses that are largely dependent on vascular nitric oxide bioavailability (for acetylcholine) or arachidonic acid metabolism (for hypoxia), respectively. The use of sodium nitroprusside, by contrast, allows for an estimation of vascular smooth muscle responsiveness to exogenous nitric oxide stimulation. These indices were also included as they represent some of the most commonly collected estimators of changes to vascular reactivity under conditions of altered cardiovascular disease risk profiles and may allow for maximum utility in this regard in terms of comparisons to existing data or data between research laboratories/groups.

The presentation of VHI in the present study does not include indices of vasoconstrictor responses (e.g., phenylephrine, endothelin-1). This decision was made after extensive consideration and reflects the variability in the literature and outcomes associated with different models of disease risk. While there is evidence that vasoconstrictor responses to an exogenous challenge can be impacted by chronic disease risk conditions (Stapleton et al., 2008; Lemaster et al., 2017b), it is far from a consensus opinion and can be extremely model-dependent, especially when compared to the monumental amount of previous evidence demonstrating the impairment to dilator reactivity in both the cerebral and skeletal muscle circulations that are well correlated with outcome risk severity (Stapleton et al., 2008; Benincasa et al., 2022; Zhang, 2022).

While the above metrics address alterations in vascular reactivity with changes in the risk severity, the mechanics of the vascular wall, particularly the progressive loss of wall distensibility, is considered one of the key changes to vascular structure and function associated with a poor health outcome. To that end, the slope (β) of the circumferential wall stress versus strain relationship was included as an optimal parameter for the calculations of VHI (Effros et al., 1981). This parameter has been very well correlated with the cardiovascular and cerebrovascular disease risk severity for many years (Laurent et al., 2006; Lacolley et al., 2020; Laurent et al., 2022) and is a critical component to the determination of VHI. Through its inclusion, we now can account for the increase in vessel stiffness with elevated risk (or following imposed interventions) and we have a critical marker of vascular mechanics at the individual vessel level of resolution.

The final parameter that was included in the calculations of VHI was that of skeletal muscle or cerebral cortex microvessel density (MVD). From a purely conceptual perspective, this adds insight and information from the “vascular network” level of resolution and speaks directly to the importance of vascularity/capillarity within the tissue under the spectrum of experimental conditions. The existing literature is replete with examples of how MVD, determined using multiple methodologies, changes with elevated disease risk and following therapeutic interventions (Liang et al., 2019; Paavonsalo et al., 2020; Wong et al., 2022). The ability to include data and insight into both the pro-angiogenic and rarefactory stimuli under an array of experimental conditions is critical for assessing the overall health and functional outcomes of the tissue. Further, it also provides direct evidence for the integrated processes of mass transport and exchange at the level of the blood-tissue exchange (BTEX) unit in both skeletal muscle and the brain (Mason McClatchey et al., 2017; McClatchey et al., 2017). Providing insight at this more encompassing level of spatial resolution is a central aspect of the VHI and improves its utility significantly beyond that of data pertaining to individual vessel function alone.

Taken together, the indices included in the VHI metric provide for multiple measures of vascular reactivity, and measures of vascular wall mechanics and microvascular network structure. While the 5-parameter VHI incorporates all these measures, the 3-parameter measure has the benefit of being simpler to use, with somewhat easier data collection requirements, and retains the multi-scale benefit of the 5-parameter calculation. In addition, the outcomes from both the 3- and 5-parameter calculation are extremely consistent and compare very favorably to each other in terms of interpreting vascular health/dysfunction severity. From the perspective of criterion validity, plasma biomarkers that have been well-established markers of chronic metabolic disease severity, plasma insulin (Figure 6) and TNF-α (Figure 7) concentrations, demonstrated a strong negative correlation with VHI in OZR under untreated control conditions and reflected appropriate changes as a result of the imposed interventions. Minimal correlation was demonstrated with VHI in healthy LZR.

Vascular dysfunction associated with the progression of metabolic disease and other conditions of elevated cardiovascular disease risk represents a diverse group of impairments to vascular tone, wall mechanics, and network geometry. The use of a singular integrated measure that can capture these impairments while accounting for age and allowing for the pooling of data is crucial in the pursuit of more focused, innovative approaches to studying vascular dysfunction in the animal models. In the results of the present study, the VHI clearly indicates that the decline in integrated microvascular structure and function with the progression of metabolic disease in male OZR was significant, multi-factorial, and was consistent over multiple years, multiple cohorts of animals, and—perhaps most importantly—over multiple groups of investigators collecting the data/results. While the contributing mechanisms for the shift in vascular reactivity (Benincasa et al., 2022; Zhang, 2022), vascular wall mechanics (Lacolley et al., 2020; Laurent et al., 2022) and microvessel density (Paavonsalo et al., 2020; Wong et al., 2022) have been extensively presented and discussed elsewhere, this speaks directly to the discriminant validity of the VHI metric and is also evident in Figures 2C, 3B,C, 4B.

As summarized in Table 6, regardless of 3- or 5-parameter calculation, in the skeletal muscle or cerebral vasculature, the resulting VHI presented clear and reproducible statistical differences between LZR and OZR. These results were not only present at the appropriate ages, depending on disease severity, they were also present to the appropriate extent at the different ages (i.e., differences at 10 weeks of age were not as pronounced as differences at 17 weeks of age). Further, intervention with chronic treatment with captopril, an anti-hypertensive agent that works through inhibition of angiotensin converting enzyme resulted in a significant, appropriately delayed, improvement to VHI that reflected previously demonstrated improvements to reactivity (Frisbee, 2005; Frisbee et al., 2018), wall mechanics and MVD.

### Applications of the metric

One of the most fundamental aspects of introducing a new metric for assessing the status of a physiological system function is determining how it can be applied most meaningfully. The development of the VHI, both the 3- and 5-parameter calculations, allows for a simple and accurate determination of the relative vascular health profile in an animal model as well as the determination of statistically significant differences in integrated vascular health outcomes between animal cohorts undergoing different interventions. In addition, the use of VHI allows for linking of datasets across time, experiments and research groups/investigators which can make comparisons of outcomes both more manageable and accessible. While VHI, taken in conjunction with other established disease risk scores and relevant biomarkers, can be used to analyze relationships between vascular health and the development of chronic pathological states, sexual dimorphism, chronic interventions, etc., it also has the ability to identify potential subgroups within analyzed datasets. Ultimately, this may have the potential to better understand disease etiology and development from the perspective of vascular dysfunction.

## Data Availability

The original contributions presented in the study are included in the article/supplementary materials, further inquiries can be directed to the corresponding author.
